# Crystal Structures of the Viral Protease Npro Imply Distinct Roles for the Catalytic Water in Catalysis

**DOI:** 10.1016/j.str.2013.04.003

**Published:** 2013-06-04

**Authors:** Thomas Zögg, Michael Sponring, Sabrina Schindler, Maria Koll, Rainer Schneider, Hans Brandstetter, Bernhard Auer

**Affiliations:** 1 Department of Molecular Biology, University of Salzburg, Billrothstraße 11, 5020 Salzburg, Austria; 2 Austrian Centre of Industrial Biotechnology, Petersgasse 14, 8010 Graz, Austria; 3 Institute of Biochemistry, University of Innsbruck, Peter-Mayr-Straße 1a, 6020 Innsbruck, Austria

## Abstract

Npro is a key effector protein of pestiviruses such as bovine viral diarrhea virus and abolishes host cell antiviral defense mechanisms. Synthesized as the N-terminal part of the viral polyprotein, Npro releases itself via an autoproteolytic cleavage, triggering its immunological functions. However, the mechanisms of its proteolytic action and its immune escape were unclear. Here, we present the crystal structures of Npro to 1.25 Å resolution. Structures of pre- and postcleavage intermediates identify three catalytically relevant elements. The trapping of the putative catalytic water reveals its distinct roles as a base, acid, and nucleophile. The presentation of the substrate further explains the enigmatic latency of the protease, ensuring a single in *cis* cleavage. Additionally, we identified a zinc-free, disulfide-linked conformation of the TRASH motif, an interaction hub of immune factors. The structure opens additional opportunities in utilizing Npro as an autocleaving fusion protein and as a pharmaceutical target.

## Highlights

• Putative catalytic water reveals distinct roles as a base, acid, and nucleophile
• The structural mechanism explains a single in *cis* cleavage
• The bimodular architecture reflects proteolytic and immunological functions
• The structure provides two orthogonal targets for therapy

## Graphical Abstract

**Figure:**
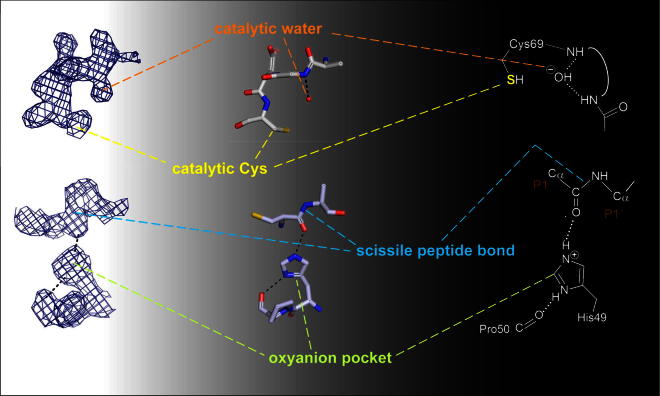

## Teaser

The pestivirus protease Npro abolishes host cell antiviral defense. Structures by Zögg et al. reveal residues that act as nucleophiles and as oxyanion pockets. The trapped catalytic water has distinct roles as base, acid, and nucleophile, and the substrate binding mode explains the single in *cis* cleavage.

## Introduction

Pestiviruses such as bovine viral diarrhea virus (BVDV) are a considerable cause of livestock disease and pathology (Peterhans and Schweizer, 2010). This versatile viral family shows a broad spectrum of strain-specific cytopathogenicity, virulence, infection modes, and persistence among their hosts comprising cattle, swine, sheep, and wildlife ruminants. Four main viral species, BVDV-1 and BVDV-2, classical swine fever virus, and boarder disease virus, were the focus of previous studies; they were distinguished based on sequence homology and antibody cross-reactivity, but further distinct species such as BVDV-3/HoBi-like pestiviruses with atypical behavior in immunological testing have been isolated (Schirrmeier et al., 2004; Ståhl et al., 2010).

The viral single-stranded positive sense RNA codes for 12 proteins translated as a single polypeptide chain. The nonstructural protein Npro (N-terminal protease) is the very first protein to be synthesized, releasing itself from the nascent polypeptide chain via a single, autoproteolytic cleavage event (Wiskerchen et al., 1991). Detached Npro is proteolytically inactive but acts as a viral immediate effector to modulate the host cell’s antiviral defenses (Ruggli et al., 2003). Mutagenesis or deletion of Npro creates attenuated viral strains but has no impact on virulence, viral replication, and protein synthesis (Mayer et al., 2004; Seago et al., 2010; Tratschin et al., 1998). Npro is thought to suppress the production of antiviral interferon (IFN)-α/β via interfering with interferon regulatory factor (IRF) 3 and IRF7 signaling, explaining this attenuated phenotype (Fiebach et al., 2011; Hilton et al., 2006; Ruggli et al., 2005, 2009). This Npro-mediated immune tolerance within infected host cells is complemented by the activities of a second viral immunomodulatory protein, E^rns^ (Schneider et al., 1993). This ribonuclease degrades extracellular RNA otherwise eliciting Toll-like-receptor-mediated IFN-α/β release through noninfected cells (Krey et al., 2012; Peterhans and Schweizer, 2013).

The conserved Glu22-His49-Cys69 triad has been invoked for catalysis, resulting in the assignment of Npro to a new subfamily of cysteine proteases, C53 (Rawlings et al., 2012; Rümenapf et al., 1998). The proteolytic self-cleavage occurs C terminally of residue 168 and is widely independent of the amino acids present in the primed substrate binding sites; only proline is not tolerated at the P1′ position (Achmüller et al., 2007; Rümenapf et al., 1993). This remarkable property makes Npro attractive for biotechnological applications as a self-releasing N-terminal fusion tag, generating authentic N termini for the respective target proteins (Achmüller et al., 2007; Kaar et al., 2009; Ueberbacher et al., 2009).

Physical interactions with and relocation of IRF3, IRF7, and the NF-κB inhibitor IκBα indicate multiple immunological interference points (Doceul et al., 2008; Fiebach et al., 2011; Hilton et al., 2006). Additionally, the interaction with the antiapoptotic molecule Hax1 suggests a putative function in the survival of infected cells (Johns et al., 2010). Several of these immunologically relevant interactions were mapped to the zinc binding TRASH motif. As mutations of zinc-coordinating residues abolished both zinc binding and IRF3 interactions, zinc binding was proposed to be essential for these protein-protein interactions (Szymanski et al., 2009). To elucidate the intriguing enzymatic and binding properties, we set out to structurally investigate this bifunctional protein.

Here, we present crystal structures of Δ(2–21)Npro from a BVDV-3/HoBi-like strain to 1.25 Å resolution, revealing a two-domain structure. The three-dimensional structure rationalizes the enigmatic single in *cis* cleavage. We provide mechanistic insight into the proteolytic assembly by describing a substrate-like, unprocessed P1′-site reaction intermediate. Here, a solvent molecule trapped at the active site offers a consistent reaction mechanism highlighting distinct roles of the catalytic water. A product-like conformation resulting from cleavage further delineates Npro’s proteolytic reaction mechanism, revealing three distinct structural elements in catalysis.

## Results

### Determination of a Minimal Npro Crystallization Construct

To identify a minimal proteolytically functional Npro construct suitable for crystallization, we evaluated N- and C-terminally truncated variants for their expression, folding, solubility, and proteolytic activity. We confirmed previous findings that deletion of the 21 N-terminal amino acids did not alter proteolytic activity while further truncation was detrimental (Hilton et al., 2006; Ruggli et al., 2009; Rümenapf et al., 1998). The peptide sequence following the C terminus of Npro did not affect folding and could be either replaced or removed. C-terminal truncation variants lacking up to four amino acids expressed well but were unstable.

From these analyses, we chose Δ(2–21)Npro_6His_ as the best-suited construct. It lacks 21 N-terminal amino acids and carries a C-terminal hexahistidine tag extension for purification purposes (Achmüller et al., 2007). This tag was autoproteolytically removed following in vitro folding from inclusion bodies (IBs), resulting in autoprocessed Δ(2–21)Npro. The construct from BVDV-3 showed highest solubility when compared to orthologous strains and thus was used for crystallographic analysis. In order to compare this postcleavage, product-like Npro with a precleavage, substrate-like variant, we produced an S169P mutant Δ(2–21)Npro S169P_6His_, as proline in P1′ was shown to prevent autoproteolysis (Achmüller et al., 2007). Indeed, this construct remained uncleaved.

### Overall Structure of Δ(2–21)Npro

To elucidate the detailed mechanism of the single autoproteolytic cleavage, we determined the structures of Δ(2–21)Npro and Δ(2–21)Npro S169P_6His_ to 1.5 Å and 2.0 Å resolution, respectively (see Table 1).

**Table 1:** Data Collection and Refinement Statistics

Construct	Δ(2–21)Npro	Δ(2–21)Npro S169P_6His_	Δ(2–21)Npro 146–150_5His_	Δ(2–21)Npro + Hg	Δ(2–21)Npro + Ir	Δ(2–21)Npro S169P_6His_ + AS	Δ(2–21)Npro at pH 3
PDB entry	3zfn	3zfo	3zfp	3zfq	3zfr	3zfu	3zft
Additive	—	—	—	mercury	iridium	ammonium sulfate	—
Resolution range (Å)	28.0-1.5	43.0-2.0	20.5-1.25	10-2.7	20.5-1.8	30.5-1.8	10.0-1.8
Space group	P2_1_	P2_1_	P2_1_	P2_1_	P2_1_	P2_1_	P2_1_
Molecules in ASU	1	1	1	1	1	1	1
Unit cell dimensions							
a	42.39	43.33	42.39	41.64	41.77	43.15	41.83
b	41.16	41.05	41.04	41.23	41.07	41.07	41.19
c	44.30	46.25	44.51	43.21	43.56	46.03	44.37
beta	115.38	98.95	115.31	114.15	114.60	98.95	115.00

**Data Collection and Refinement**							

Measured reflections	78,576	42,374	141,513	14,646	43,476	31,301	44,184
Unique reflections used	21,445	10,911	37,962	4,228	12,457	14,102	12,285
Completeness (%) (outer shell)	96.5 (95.3)	98.9 (85.3)	98.9 (97.4)	100 (100)	98.7 (91.3)	89.6 (93.7)	95.4 (93.8)
Multiplicity (outer shell)	3.7 (3.7)	3.9 (3.5)	3.7 (3.7)	3.5 (3.5)	3.5 (3.3)	2.2 (2.2)	3.6 (3.6)
R_pim_ (outer shell)	0.029 (0.125)	0.089 (0.341)	0.028 (0.297)	0.086 (0.284)	0.046 (0.401)	0.054 (0.282)	0.050 (0.593)
R_merge_ (outer shell)	0.047 (0.209)	0.151 (0.547)	0.047 (0.489)	0.137 (0.453)	0.074 (0.617)	0.071 (0.369)	0.082 (0.976)
Mean (<I>/sd <I>) (outer shell)	15.0 (5.2)	26.7 (3.2)	12.4 (2.6)	7.6 (2.8)	10.8 (2.0)	6.9 (2.1)	11.3 (1.2)
R_crys_ (R_free_)	0.17 (0.24)	0.20 (0.25)	0.16 (0.21)	0.21 (0.28)	0.18 (0.23)	0.20 (0.24)	0.19 (0.27)

**Rmsd from Ideal Geometry**							

Bond length	0.021	0.018	0.026	0.010	0.020	0.020	0.013
Bond angles	2.06	1.89	2.51	1.27	2.02	2.12	1.45
Ramachandran statistics (%)	99.3/0.7	99.3/0.7	97.9/2.1	97.2/2.8	99.3/0.7	99.3/0.7	94.1/1.4
Allowed/outliers							
Ligands							
1-thioglycerol	+	+	+	+	+	+	+
Chloride	+	+	+	−	−	−	+
Hydroxide	−	+	−	−	+	−	−
Other ions	−	−	−	mercury	2 iridium	sulfate	−

Rmsd, root-mean-square deviation.

**Figure 1:**
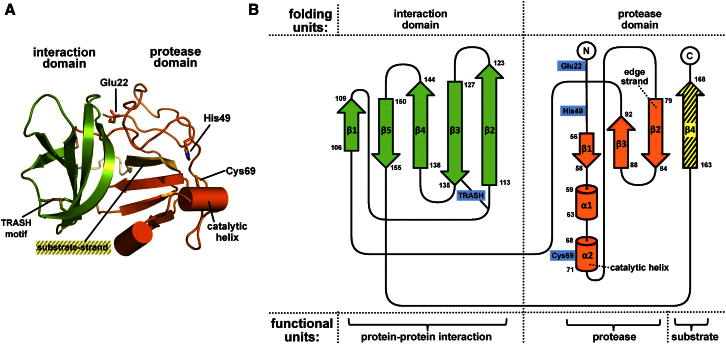
Overall Structure and Topology of N-Terminal Protease (A) Overview of the overall fold. Npro reveals a two-partite domain arrangement with the protease domain indicated in orange and the interaction domain in green. Selected side chains are represented as sticks. (B) The topology diagram highlights the assignment of three functional units. The protease domain is composed of the actual protease including the catalytic helix plus a β4 strand (yellow, hatched) acting as a substrate recognized by the β2-edge strand. The topology diagram was prepared with TopDraw software (Bond, 2003).

The overall structure revealed two compacted folding units: a protease domain (orange) and an interaction domain (I-domain, green) (see Figure 1A). Functionally, the protease domain can be further divided into the actual protease (orange) and the substrate strand (yellow) (Figure 1B).

Spanning residues Phe106 to Val155, the I-domain adopted a five-stranded antiparallel β sheet with Greek key architecture and was tethered to the protease domain via a hydrophobic surface anchored around Trp154. The proline-rich protease domain contained the complete catalytic environment and was composed of a four-stranded antiparallel β sheet core, a significant portion of random-coil elements, and two short helices, including the distorted catalytic helix α2 (Figure 1B). The strand β4 acted as substrate and was contributed by the C-terminal residues 163–168. It passed through the core of the molecule, which explains why truncations at the C terminus render the protein unstable. The linker region between the I-domain and the substrate strand was surface exposed, representing a major hydrophobic surface patch.

No similarity could be detected for the overall architecture of the protease domain using the web servers TopSearch and DaliLite (Z scores below 2) (Frank et al., 2010; Holm et al., 2008). It is stabilized by charge interactions, which are highly conserved among different homologs. The buried side chains of Arg51 and Asp92 interact with the backbone amides and carbonyls of Tyr25, Gly52, Glu53, Tyr93, and Gly95, while salt bridges are found between Glu22 and Arg100 as well as Lys84 and Tyr90. Side-chain hydrogen bonds between Asn73 and Gln91 further complement the domain interaction.

There were only minor deviations between the substrate- and product-like structures including their ligand-bound forms described below, mainly arising from alternate loop and side-chain conformations. While the proteins crystallized in the same space group P2_1_ with similar cell constants, their monoclinic β angle diverged for up to 16°. However, the largest backbone C_α_ root-mean-square deviation was <0.7 Å, as calculated by the TopMatch superposition server (Sippl and Wiederstein, 2012). Npro molecules from different viral strains are likely to share the same structure, as nonconservative amino acid exchanges occur only at surface-exposed residues. Altered surface charges nevertheless suggest adaptation to specific host interactions.

### Δ(2–21)Npro Reveals Three Structural Elements in Catalysis

Based on mutational studies, Npro was previously reported to harbor a catalytic triad consisting of Glu22, His49, and Cys69 (Rümenapf et al., 1998). Our experimental Δ(2–21)Npro structure confirmed important roles for Cys69 and His49. By contrast, Glu22 was ∼20 Å distant from the active site and rather stabilized the structure through salt-bridge formation with Arg100; it cannot participate in the catalytic reaction (Figure 1A). Mutations of Glu22 thus destabilize the protein, explaining the concomitant reduced or abolished catalytic activity and IFN induction (Hilton et al., 2006; Ruggli et al., 2009; Rümenapf et al., 1998).

**Figure 2:**
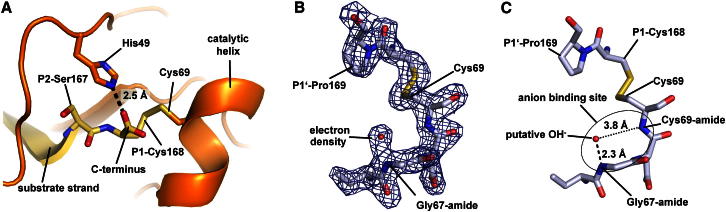
Identification of an Anion Binding Site (A) Close-up view of the catalytic environment including Cys69 and the substrate strand (yellow) in cleaved, product-like Δ(2–21)Npro. The catalytic Cys69 is trapped in a disulfide bond with the P1-Cys168 side chain (see also B). His49 stabilizes the carboxylate of the C terminus. (B) Identification of a defined density close to the Gly67-amide in uncleaved, substrate-like Δ(2–21)Npro S169P_6His_ (gray). (C) The bound entity at a 2.3 Å distance from the Gly67-amide is fully consistent with a negatively charged hydroxide ion, identifying this motif as hydroxide binding pocket.

Consistent with its proposed role as nucleophile in catalysis, we found the first catalytic element Cys69 to be located close to the scissile peptide bond on a distorted α helix. Intriguingly, Cys69 was locked in a disulfide with the P1-residue Cys168, reflecting a ∼3 Å displacement of the Sγ from the position where it could attack the scissile peptide bond (Figure 2A).

As a second element, we propose that the catalytic His49 serves as oxyanion pocket (depicted in the product-like structure in Figure 2A); His49 is positioned at the opposite side of the substrate strand and is therefore too distant to assist in Cys69 deprotonation, similar to caspases. Rather, His49 polarizes the scissile peptide bond carbonyl oxygen through hydrogen bonding and thus supports formation of the tetrahedral transition state intermediate. The imidazole ring is further aligned and polarized by the interaction of its Nδ with the Pro50 carbonyl.

The third element spans Lys66 to Arg70 and harbors a characteristic anion binding site. This structural motif is highly conserved among different viral strains and is anchored by Pro64 and the Cys69-Cys168 disulfide. Importantly, in the substrate-like Δ(2–21)Npro S169P_6His_ structure (3ZFO), we found that the Gly67 and Cys69 amides formed a partially charged pocket occupied by a solvent molecule, as reflected by a well-defined electron density (Figure 2B). In the following, we provide several lines of evidence that determine the identity of this density.

We could exclude covalent modifications of the Gly67 amide, such as methylation, as the density disappeared upon conformational rearrangement of the binding pocket (compare 3ZFU). While the scattering power of approximately 7–10 electrons suggests that N/O/F/Na^+^/Mg^2+^ occupy the site, its coordination by partially positive amides of Gly67 and Cys69 selects for negatively polarized ligands, excluding cations as well as ammonium. A fluoride ion appears highly unlikely due to its rare occurrence and untypical coordination, leaving the ubiquitous molecules H_2_O and OH^−^ as remaining candidates. The following observations allow us to discriminate between these two molecules.

First, the solvent molecule reveals an untypically short bond distance to the Gly67 amide of 2.3 Å, indicative of a short strong hydrogen bond (SSHB, aka low-barrier hydrogen bond) typically ranging from 2.3 Å to 2.5 Å (Cleland, 2000). Such bonds form between hydrogen bond donors and acceptors with similar pKa values of the respective H-carrying species. This is strongly suggestive of a SSHB formed between the Gly67 amide hydrogen (pKa of ∼15) and an OH^−^ (pKa of the corresponding H_2_O is ∼15) and not an H_2_O (pKa of the corresponding H_3_O^+^ is ∼−1.7) (Atkins and de Paula, 2010).

Second, we observe the solvent molecule at the very N terminus of a distorted helix. The dipole located at the N-terminal helix edge amounts to an ∼0.5(+) partial charge, attracting negatively charged counter ions such as OH^−^ (Aqvist et al., 1991, Harris and Turner, 2002).

Third, the positively charged side chains of Arg70 and Lys66 contribute to stabilize the hydroxide within the anion binding site.

### The Anion Binding Site as a Trap for the Catalytic Water

**Figure 3:**
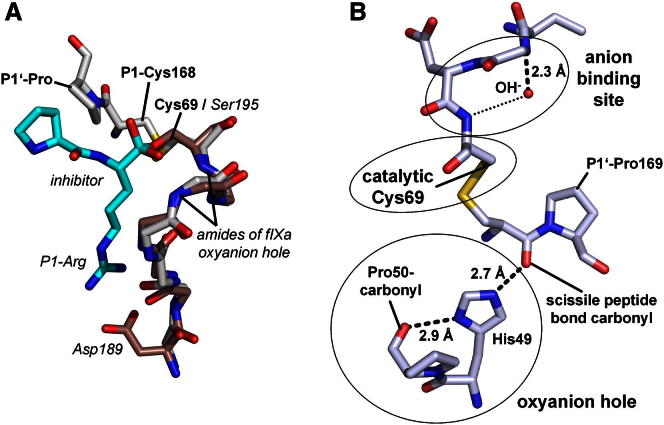
Identification and Functional Relevance of Three Catalytic Elements (A) Superposition of Δ(2–21)Npro S169P_6His_ (gray) with the tryptic serine protease human fIXa (fIXa, brown, italics) including an oxyanion hole stabilizing inhibitor (turquoise, italics). This comparison reveals remarkable structural similarity between the motifs, despite functional differences (B). (B) Three catalytic elements are highlighted by circles: the catalytic Cys69, the His49 acting as oxyanion hole, and the anion binding site (ABS). The suggested underlying reaction mechanism is described in detail in Figure 4.

Regarding its functionality, a remarkable sequence similarity of this binding motif (GDC_69_) with the corresponding residues in (chymo-)trypsin-like serine proteases such as human coagulation factor IXa (fIXa; GDS_195_) was pointed out previously (Rümenapf et al., 1998). In these serine proteases, GDS_195_ forms the oxyanion hole necessary for the catalytic reaction. Superimposing this motif from substrate-like Δ(2–21)Npro S169P_6His_ onto active-site-inhibited fIXa (hfIXa, Protein Data Bank [PDB] entry 2WPI) revealed virtually identical conformations (Figure 3A) (Zögg and Brandstetter, 2009). At first glance, this similarity might lead to the conclusion that this structural motif acts as oxyanion hole for Npro. However, such an interpretation as a classical oxyanion binding pocket is inconsistent with several geometric restraints that are imposed by the structure. The substrate strand β4 with its P6–P1 residues is rigidly anchored to the edge strand β2 and His49 by a hydrogen bond network both before and after cleavage (Figures 1, 2A, and 3B). The scissile P1 carbonyl group would need to detach from His49, flip 180°, and move ∼5 Å to reach the anion binding site (Figure 3B).

Additionally, the position of the anion binding site N terminal to a helix has altered its biochemical properties as compared to the situation in serine/cysteine proteases. The helix dipole causes an electrostatic lensing effect, breaking the symmetric binding to the Gly67 and Cys69 amides (Figure 2C).

**Figure 4:**
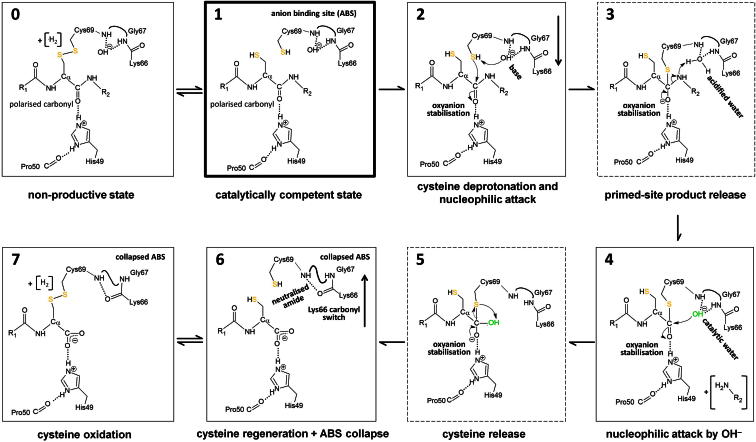
Proposed Catalytic Reaction Mechanism for Npro (0 and 1) The proteolytically competent Npro shows a protonated Cys69 as well as a hydroxide-loaded anion binding site (1). His49 polarizes the scissile peptide bond carbonyl for nucleophilic attack. This situation is in equilibrium with a nonproductive state (0) where the Cys69 forms a disulfide with P1-Cys168. (2) The reaction is initiated by deprotonation of Cys69, which is helped by the hydroxide acting as a base, but may also occur spontaneously. Cys69 then approaches and attacks the electrophilic carbon. The resulting tetrahedral intermediate transition state is stabilized by His49 acting as oxyanion pocket. (3) The water in the anion binding site is short-lived. Immediately, a proton is transferred to the amide for primed-site product release; the water thus acts as an acid. (4) In a second step, the hydroxide can directly perform the nucleophilic attack, acting as activated catalytic water. (5) The catalytic Cys69 is released upon protonation by the newly formed C terminus, regenerating the active site. (6 and 7) The negatively charged C terminus tends to expel the hydroxide, resulting in an ABS collapse. Upon proximity-induced spontaneous oxidation, the catalytic Cys69 forms a disulfide bond with the P1-Cys168 side chain. For preparation of this figure, Chemsketch software was used (ACD/ChemSketch, 2012). See also Figure S1.

While we cannot exclude major conformational rearrangements, all of our structural data indicate that the anion binding site cannot serve as oxyanion hole but rather serves to accumulate an (activated) water (Figure 2), consistent with the catalytic water, as described in Figure 4. It sheds light to the origin and versatile roles of the “catalytic water” and the biophysical properties of its binding site, reflected by the available crystal structures.

### The ABS Is an Electrostatic Ion Trap Regulated by Charge, Ionic Strength, and pH

**Figure 5:**
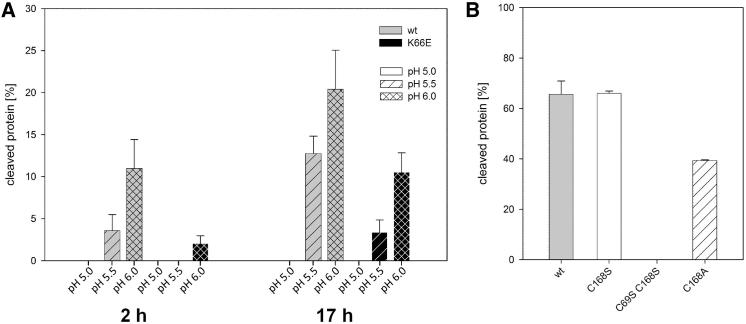
Catalytic Activity and pH Dependence of Wild-Type and Mutant Npro (A) Time course of self-processing by Δ(2–21)Npro_6His_ (gray bars) and Δ(2–21)Npro K66E_6His_ (black bars) at pH 5.0 (clear), pH 5.5 (hatched), and pH 6.0 (crossed). The activity strongly increases for all constructs with increasing pH. As compared with wild-type Npro, the K66E mutant exhibits strongly decreased activity. (B) The comparison of self-processing by Δ(2–21)Npro_6His_ with several mutants reveals a substrate preference in the P1 residue 168 for cysteine (gray)/serine (white) over the smaller alanine (hatched), while the control double mutant lacks any proteolytic activity. Data are represented as mean ±SEM of triplicate measurements.

Two positive charges at position 66 and 70 are strictly conserved. While there is no obvious requirement of this conservation for the protein structure, it appeared plausible that the positive charge is crucial to additionally favor OH^−^ binding to the anion binding site (ABS) via long-range electrostatic interactions. We therefore tested the proposed electrostatic contribution by engineering the charge-reversing mutation K66E. We indeed observed an almost 50% decreased activity at otherwise identical time points and pH values, consistent with the proposed electrostatic contribution to hydroxide accumulation (Figure 5A).

When compared to water, an OH^−^ is more likely to be affected by changes in the salt concentration through electrostatic shielding (Lanyi, 1974). Correspondingly, low salt should favor OH^−^ binding while high salt should force its release, consistent with our observations. Low-salt conditions enabled the loading of hydroxide into an otherwise unoccupied site (<50 mM NaCl; PDB entry 3ZFR compared to 3ZFN), while high-salt conditions extracted it from an occupied site (0.2 M ammonium sulfate; PDB entry 3ZFU compared to 3ZFO).

An even stronger effect on the potential hydroxide may be inferred by changing the pH. When compared to the experiment above, the anion binding site was unoccupied when crystals were exposed to pH 3, indicating the importance of hydroxide availability (PDB entry 3ZFT). This pH-dependent hydroxide reduction correlated with the reduction of enzymatic activity at pH ≤ 5.5 (Figure 5A).

**Figure 6:**
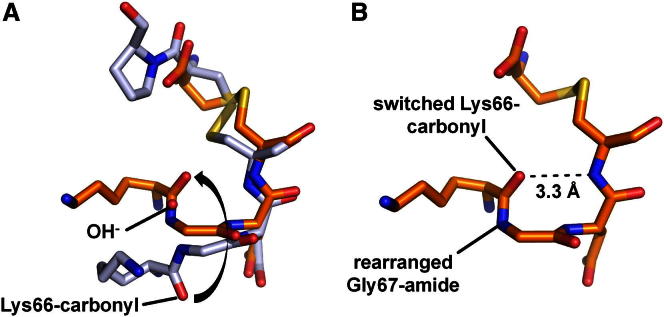
Collapse of the Anion Binding Site (A) The intact hydroxide-loaded ABS (gray) is destabilized by the product C terminus as well as by high salt. During the ABS collapse (orange), the Lys66 carbonyl performs a 180° switch, distorting the hydroxide binding pocket. (B) In the collapsed form the Lys66 carbonyl oxygen neutralizes the Cys69 amide through a hydrogen bond.

The unloading of the anion binding site was accompanied with a conformational distortion of the binding motif: a 180° flip of the neighboring Lys66 carbonyl resulted in a rotation of the Gly67 amide (Figures 6A and 6B). In consequence, the Cys69 amide hydrogen bonded with the flipped Lys66-carbonyl in a 1–4 tight turn geometry extending the helical structure and thus abolishing hydroxide stabilization. Interestingly, at intermediate salt concentration (∼100 mM NaCl), the substrate- and the product-like structures differ as the latter lacks the bound solvent (PDB entries 3ZFO and 3ZFN). This divergence is likely due to the additional negative charge contributed by the P1 C terminus in the product-like form, which tends to expel any hydroxide ion. At very low salt conditions, we observed a more symmetric binding geometry of a solvent molecule in the product form (2.5 Å and 2.9 Å to the Gly67 and Cys69 amides, respectively; entry 3ZFR). This geometry points to a mixed occupancy by a hydroxide and a neutral water molecule, consistent with the negative charge of the C terminus.

### Δ(2–21)Npro_6His_ Performs a Single In *cis* Cleavage Mediated by its Characteristic Substrate Binding Mode

**Figure 7:**
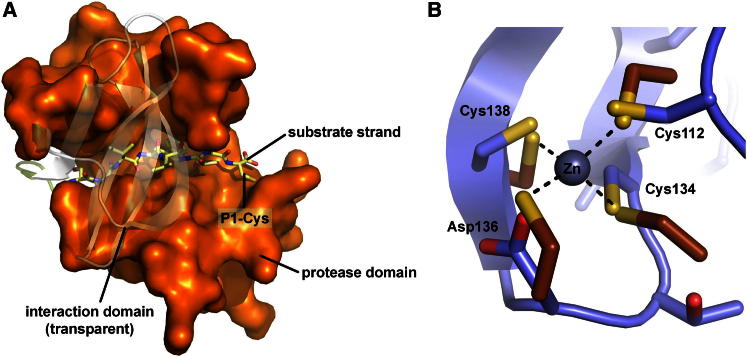
Substrate Recognition and Zinc Binding (A) The geometry of substrate strand binding in Npro explains the single proteolytic in *cis* cleavage. The C-terminal residues depicted as yellow sticks pass between the protease (orange surface) and the interaction domain (transparent cartoon representation). Therefore, the substrate β strand remains trapped within the molecule and no further substrate can bind. This structural arrangement ensures a single in *cis* cleavage event. (B) Comparison with a zinc-loaded TRASH motif structure (brown, including the zinc ion) shows that geometric restraints allow Npro to coordinate zinc in a typical tetrahedral coordination. See also Figure S2.

As Npro releases itself from the growing polypeptide chain, the protein’s own C-terminal residues Ile163-Cys168 act as the nonprimed recognition residues that bind to the respective substrate binding pockets. This substrate strand recognition occurs via canonical antiparallel β sheet extension at the β2 edge strand (Figure 1B). Given its covalent linkage to the protein polypeptide chain, the nonprimed substrate strand cannot dissociate after proteolytic cleavage; instead, the β4 substrate strand is embraced by the protease and the interaction domains and thus fixed to the active site (Figure 7A). This structural arrangement ensures a single cleavage event in *cis,* excluding the possibility for a new substrate to bind.

While the substrate strand binding is dominated by backbone interactions, we found a substrate preference at P1 for cysteine and serine over the smaller side chain alanine (Figure 5B). Interestingly, the activities of wild-type and the C168S mutant Npro are identical, suggesting that the disulfide-intermediate Cys69-Cys168, if it were to occur before cleavage, is not rate limiting in wild-type Npro. Additional mutation of the catalytic Cys69 to serine completely inactivated Npro, confirming the role of Cys69 thiolate as a nucleophile (Figure 5B).

Finally, we investigated C-terminal truncations of Δ(165/166/167-168)Npro, which should render the S4-S1, S3-S1, and S2-S1 freely accessible to substrates. However, when tested with the S1-directed substrate (H-Cys-pNA)_2_ at a 0.1–5 mM concentration in the presence of the reducing agents β-mercaptoethanol, DTT, TCEP, and 1-thioglycerol from 0 to 20 mM, these truncation variants showed no detectable substrate turnover.

### The I-Domain Harbors a TRASH Motif Involved in Protein Interaction

The I-domain, spanning residues 106–155, folded into a five-stranded antiparallel β sheet with β2–β5 forming a Greek key motif (Figure 1B). Structural homology searches using TopSearch and DaliLite found weak similarities to other proteins, although they appeared functionally unrelated (*Z* scores of 3.8–4.2) (Frank et al., 2010; Holm et al., 2008; Sippl and Wiederstein, 2012). The I-domain only indirectly influences the proteolytic activity of Npro by arresting the nonprimed substrate strand β4 at the active site.

The I-domain harbored a TRASH motif formed by Cys112, Cys134, and Cys138, which along with Asp136 was proposed to coordinate a zinc ion (Ettema et al., 2003). Structurally, this motif connected the β1-β2 linker to the β3 strand (Figure 1). Single site mutations within this motif abolished both physical zinc binding and protein interaction with IRF3 and IRF7 (Fiebach et al., 2011; Ruggli et al., 2009; Szymanski et al., 2009). We determined 1.25 Å and 1.5 Å crystal structures for solubly expressed and in-vitro-folded Npro, respectively. In neither case could we observe zinc binding at the TRASH motif but disulfide bridging between Cys112 and Cys134. The mixed disulfide of the third cysteine 138 with the reducing agent 1-thioglycerol present in the protein buffer indicated oxidizing conditions during crystal growth.

While we could not incorporate zinc into the TRASH motif by soaking experiments with reducing agents and/or zinc, we performed molecular modeling studies to evaluate whether steric restraints would allow zinc coordination. Comparison of Npro with a zinc-loaded TRASH motif based on four coordinating cysteines (PDB entry 2DAS) showed that χ1 rotations of the cysteine and aspartate side chains and reorienting a surface-exposed Lys side chain were sufficient to superimpose all zinc-coordinating atoms, resulting in a tetrahedral coordination sphere with ∼2.3 Å Sγ(Cys)-Zn^2+^ distances (see Figure 7B).

There exists evidence for potential further protein interaction sites apart from the TRASH motif originating from soaking experiments. These comprise a chloride binding site and an electrostatic interaction cluster around Arg117 (see Figure S2 available online).

## Discussion

The protein interactions of Npro with the immune-relevant factors IRF3, IRF7, and Hax1 have all been mapped to the interaction domain harboring the TRASH motif. As exemplified by the Npro-Hax1 interaction, the Npro consensus sequence spans residues 106–143. This segment includes not only the TRASH motif (Cys112, Cys134, Cys138, and Asp136) but also the Arg117 subsite (see Figure S2). The crystal structure thus helps to rationalize how the I-domain reveals functionalities distinct from the protease domain (Fiebach et al., 2011; Hilton et al., 2006; Johns et al., 2010). These binding sites may further help to map binding of other molecules, such as IκBα or a synthetic poly-K(R)W(Y) material (Doceul et al., 2008; Hahn et al., 2010).

Individual C112A, C134A, C138A, as well as D136N mutations in the TRASH motif abolished its zinc coordination and partially interrupted IRF3/IRF7 interactions in the Npro homologs Alfort and BVDV-1 (Fiebach et al., 2011; Ruggli et al., 2009; Szymanski et al., 2009). These findings corroborate our modeling studies, indicating that TRASH disulfides are reversible and able to adopt a zinc binding conformation without major alterations (Figure 7B). Cysteine-carrying zinc binding sites are known to act as redox-sensitive elements often involved in regulatory processes (Wouters et al., 2010). The unexpected disulfide may point to such a function in Npro that awaits further investigation.

The protease domain harbors three catalytic elements (Cys69 nucleophile, His49, and anion binding site) in a compact fold of less than 100 amino acids. The substrate strand passing through the protein core elegantly ensures a single in *cis* cleavage.

The catalytic element His49 was originally proposed as the catalytic base present in typical catalytic triads of cysteine proteases (Rümenapf et al., 1998). Caspases, however, show a divergent role for the histidine, which, due to distance restraints, cannot assist in cysteine deprotonation but helps in product release (Fuentes-Prior and Salvesen, 2004). Given the direct interaction with the scissile peptide bond carbonyl before and after cleavage, we suggest that His49 serves as an oxyanion “hole” in Npro. Polar side chains are known to contribute to oxyanion holes (e.g., Asn in the cysteine protease papain [Ménard et al., 1991] and His in legumain, in the latter case with the help of additional backbone amides for polarization [Elfriede Dall, personal communication]).

The monovalent polarization of the scissile peptide bond by His49 will hardly render the carbonyl carbon electrophilic enough for nucleophilic attack. We propose that a complementary mechanism may assist in increasing the electrophilicity of the carbonyl carbon where the protonation of the P1′ nitrogen precedes the nucleophilic attack by the Cys69 thiolate (see Figure S1, 2b and 3b).

Several indications point to the existence of a hydroxide ion at the anion binding site. The geometric proximity of the activated water molecule to the scissile peptide bond suggests its relevance in catalysis. The proposed reaction scheme illustrates how the water can adopt distinct roles in catalysis, including base, acid, and nucleophile, Figure 4.

This work has manifold impact. Biotechnologically, the structure allows exploitation of Npro as a fusion partner that enables exact autocatalytic trimming. The structure furthermore offers two orthogonal target points for treating infected animals by hitting the proteolytic machinery and the immune-suppressive interaction sites, respectively.

## Experimental Procedures

### Cloning and Expression of Native Protein

Cloning, expression, and purification of Npro from the pestiviral strain BVDV-3/HoBi followed the protocols for Npro variants of classical swine fever virus described previously (Achmüller et al., 2007). DNA encoding Npro derived from BVDV-3 was synthesized (Sloning). Due to incorporation of an SpeI restriction site, the amino acid sequence carries a G166T mutation. Following deletion of residues 2–21 by site-directed mutagenesis, Δ(2–21)Npro was cloned into the pET30b expression vector (Novagen) including a C-terminal 6His-tag (S_169_VDKLAAALEHHHHHH_184_, a ten-amino-acid linker connects Npro with a His_6_-tag). By applying site-directed mutagenesis to Δ(2–21)Npro_6His_, Δ(2–21)Npro S169P_6His_, Δ(2–21)Npro K66E_6His_, Δ(2–21)Npro C168S_6His_, Δ(2–21)Npro C168A_6His_, and Δ(2–21)Npro C69S C168S_6His_ were generated through replacement of the respective codons. For Δ(2–21)Npro_146–150His_ intended for soluble expression, the internal R_146_EGQD_150_ sequence was exchanged with H_146_HHHH_150_ while the terminal 6His-tag was replaced by a Strep-tag (WSHPQFEK). This allowed separation from soluble, uncleaved molecules; this purification step is important because cleavage of Npro is incomplete, with a typical yield of 25%–75%.

BL21 (DE3) *E. coli* cells were transformed with these constructs and cells were grown to an optical density 600 ∼0.8 in shaking flasks at 37°C. Protein expression by addition of 1 mM IPTG mainly yielded IBs. After 4 hr, cells were harvested and prepared for cell lysis by a French press. Before solubilization in 5 M guanidinium HCl, 100 mM NaCl, 10 mM 1-thioglycerol (MTG; Sigma), and 20 mM Tris (pH 7.5), the IBs were washed twice in buffer supplemented with 0.5% Triton X-100.

### Protein In Vitro Folding, Purification, and Crystallization

In vitro folding was carried out in 20 mM Tris (pH 7.5), 5% glycerol, 100 mM NaCl, and 10 mM MTG by rapid dilution. During in vitro folding, Δ(2-21)Npro_6His_ autoproteolytically removed the 6His-tag, which allowed separation of processed from uncleaved and likely misfolded molecules through a negative Ni-NTA purification step. Samples were then concentrated in a stirred ultrafiltration cell (Amicon) equipped with a regenerated 10 kDa MWCO ultrafiltration membrane. Subsequent ion exchange (MonoS 5/5 HR column, GE Healthcare, elution with a 500 mM NaCl gradient) and size exclusion chromatography (Superdex 75, 10/300 GL, GE Healthcare, in 20 mM Tris [pH 7.5], 5% glycerol, 100 mM NaCl, and 1 mM MTG) further increased protein purity. Due to lack of proteolysis in Δ(2–21)Npro S169P_6His_, the negative Ni-NTA purification step was skipped.

To purify soluble Npro_146-150His_ a native Ni-NTA affinity chromatography was performed after cell disruption in 20 mM Tris (pH 7.5), 5% glycerol, 1 M NaCl, 1 mM MTG. The negative purification was carried out on a Strep-Tactin column (GE Life Sciences), followed by desalting (PD-10, GE Healthcare), ion exchange (HiTrap SP FF, 1 ml CV; GE Life Sciences), and size exclusion. Proteins were concentrated with Centricon centrifugal filter devices (10 kDa MWCO, Millipore) to ∼6 mg/ml and crystallized by mixing protein and crystallization solution (100 mM Na acetate [pH 8.5] and 25%–50% PEG 6000) at ratios of 1.5:1 to 2:1 in a batch under oil setup using mineral oil (Sigma, M5904). Crystals grew within 3 days to a size of approximately 0.2 × 0.2 × 0.05 mm at 20°C.

### Activity Assays

To establish complete denaturation before activity analysis, IBs were solubilized with 8 M guanidinium HCl, 10 mM MTG, and 50 mM Tris (pH 7.5) and the concentration adjusted to 1.5 mg/ml. For the pH-dependent activity determination, samples were diluted into a buffer containing 15% glycerol, 50 mM NaCl, 20 mM Tris, 50 mM glycine, 25 mM sodium phosphate, and 10 mM MTG (pH 3.1). After 15 hr, pH was adjusted by adding 20% of 1 M ammonium phosphate, 50 mM NaCl, and 15% glycerol with a pH of 5.0, 5.5, or 6.0. The time course of the pH-shift reaction was monitored by SDS-PAGE electrophoresis of samples drawn after 2 and 17 hr. For cysteine mutant activity determination, samples were directly diluted into 30% glycerol, 100 mM NaCl, and 20 mM Tris (pH 7.5) and incubated for 15 hr at room temperature. Following Coomassie staining, gels were densitometrically analyzed with the ImageJ software (Schneider et al., 2012).

### Preparation of SeMet-Labeled Protein

For phasing purposes, we expressed SeMet-substituted Δ(2–21)Npro_6His_ following a protocol described previously (Van Duyne et al., 1993). In short, BL21 (DE3) cells were grown in M9 minimal medium supplemented with L-lysine, L-threonine, L-phenylalanine, L-leucine, L-isoleucine, and L-valine suppressing natural methionine biosynthesis. A total of 50 mg/l seleno-DL-methionine (Sigma) was added to the growth media right before induction with 1 mM IPTG. Cell disruption, protein purification, folding, concentration, and crystallization were performed under similar conditions as native Npro.

### Crystal Soaking, X-Ray Data Collection, and Structure Determination

Crystals were directly flash-cooled in the 100 K nitrogen cryostream for diffraction quality analysis on the X-ray home source. Data collection was carried out either in-house using a 345 mm MARdtb image plate (MAR Research, Hamburg) hooked up to a Bruker Microstar rotating copper anode system or at synchrotron sources (the MAD beamlines ID14-4 and ID19 at ESRF in Grenoble, France, and the K1.2 [EMBL X12] beamline at DESY in Hamburg, Germany).

For soaking experiments, crystals were transferred into 2–5 μl of 100 mM Na acetate (pH 8.5; for the pH 3 data set 300 mM Na acetate, pH 3.1) and 45%–60% PEG 6000 plus the respective additive [200 mM (NH_4_)_2_SO_4_ (Merck); 2 mM K_2_IrCl_6_ (Jena Biosciences) or 2 mM HgCl_2_ (Jena Biosciences)] and flash-cooled within 30–180 s.

For native Npro, X-ray diffraction data to a resolution of 1.5 Å were collected from a single crystal belonging to the space group P2_1_. We evaluated and processed diffraction images with the iMOSFLM beta-release version (Battye et al., 2011). Merging and scaling was carried out using the CCP4 suite (Collaborative Computational Project, Number 4, 1994; Evans, 2006; Winn et al., 2011).

The Npro structure was solved with a three-wavelength MAD (3W-MAD) approach from a single crystal containing SeMet protein. Data collection and refinement statistics for the three measured data sets are shown in Table 2.

**Table 2:** Data Collection and Refinement Statistics from a Single SeMet-Containing Crystal

Data Set	Peak	Inflection	Low-Energy Remote
λ (Å)	0.9737	0.9795	0.9999
Resolution range (Å)	75.0-2.5	75.0-2.7	75.0-2.6
Space group/molecules in ASU		P2_1_/8	
a/b/c		71.99/117.87/75.26	
beta		96.82	

**Data collection and refinement**			

Measured reflections	321,521	252,027	143,884
Unique reflections used	42,884	33,762	38,679
Anomalous completeness (%) (outer shell)	100 (100)	99.7 (100)	93.7 (91.2)
Anomalous multiplicity (outer shell)	3.8 (3.8)	3.8 (3.8)	1.9 (1.9)
R_pim_ (outer shell)	0.069 (0.434)	0.057 (0.257)	0.097 (0.831)
R_merge_ (outer shell)	0.115 (0.732)	0.095 (0.433)	0.100 (0.864)
Mean (<I>/sd<I>) (outer shell)	12.0 (2.6)	13.7 (4.2)	7.2 (1.4)

These data sets served as input for the 3W-MAD protocol of the Auto-Rickshaw platform (Panjikar et al., 2005). First, structure factor amplitudes were calculated with SHELXC (Sheldrick, 2010). Data to 3.2 Å were used for initial phase calculation. A total of 24 Se positions were identified by SHELXD corresponding to eight molecules in the asu and a solvent content of 48% (Schneider and Sheldrick, 2002). The correct handedness was determined with the programs ABS and SHELXE (Hao, 2004). The occupancy of the Se sites was further refined with MLPHARE (Winn et al., 2011). The program RESOLVE could identify a two-fold noncrystallographic symmetry operator (Terwilliger, 2000). Density modification, phase extension, and noncrystallographic symmetry averaging were performed with dm (Cowtan, 1994). Finally, a partial model was generated with the program HELICAP containing 773 out of 1,184 residues (Morris et al., 2004). From here, an MRSAD protocol was applied by the autorickshaw pipeline, including several phasing and building cycles, including programs PHASER, MLPHARE, SHELXE, RESOLVE, REFMAC5, and ARP/wARP (Collaborative Computational Project, Number 4, 1994; Langer et al., 2008; McCoy et al., 2007; Murshudov et al., 1997; Sheldrick, 2010; Terwilliger, 2000). The resulting model included 928 residues out of 1,184 and showed an R_crys_/R_free_ ratio of 0.36/0.43.

After manual rebuilding, model completion, and refinement in Coot (Emsley and Cowtan, 2004), one molecule of the ASU served as model for molecular replacement of the 1.5 Å native data set carried out with MOLREP (Vagin and Teplyakov, 2010). The resulting structure was refined to an R_crys_/R_free_ ratio of 0.17/0.24 using repeated manual model building and REFMAC5 refinement and served as model for structure solution of other data sets. Refinement statistics for all data sets are given in Table 1.

Model validation involved Coot, pdb-sum, and NQ-flipper (Laskowski et al., 2005; Weichenberger and Sippl, 2007), and structure superpositions were performed with the TopMatch-webserver and Coot (Emsley and Cowtan, 2004; Sippl and Wiederstein, 2012). Figures including protein structures were prepared with PyMOL (DeLano, 2005).

## Accession Numbers

Atomic coordinates and structure factors have been deposited in the Protein Data Bank with the accession entries 3ZFN, 3ZFO, 3ZFP, 3ZFQ, 3ZFR, 3ZFT, and 3ZFU.
