# Supplementary material for: Crystal Structures of the Viral Protease Npro Imply Distinct Roles for the Catalytic Water in Catalysis
Source: Structure. 2013 Jun 4;21(6):929–38. doi: 10.1016/j.str.2013.04.003 (PMC3677099; doi:10.1016/j.str.2013.04.003)
Supplement: Document S1. Figures S1 and S2 [file mmc1.pdf]

Structure, Volume *21*

Supplemental Information

Crystal Structures of the Viral Protease Npro Imply Distinct Roles for the Catalytic Water in Catalysis

Thomas Zögg, Michael Sponring, Sabrina Schindler, Maria Koll, Rainer Schneider, Hans Brandstetter, and Bernhard Auer

Inventory of Supplemental Information

Supplemental Data:

Figure S1, related to Figure 4.

Figure S2, related to Figure 7.

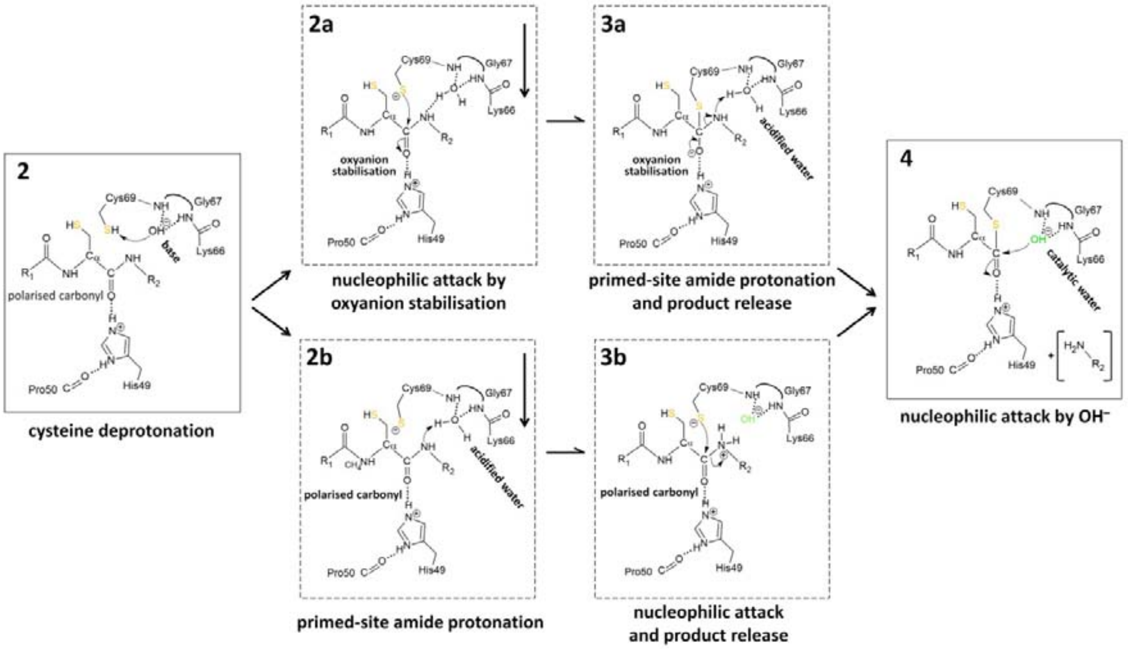

Figure S1: Alternative catalytic reaction mechanism involving amide protonation, related to Figure 4.

(**2**) Following deprotonation of Cys69, the catalytic reaction may follow two distinct ways: The classical pathway depicted in the upper 2 panels (**2a, 3a**) suggests a strong oxyanion hole contribution which allows an immediate nucleophilic attack followed by primed-site product release. Alternatively, in the lower 2 panels (**2b, 3b**), the P1’ amide is protonated by the acidified water generating a substituted ammonium. Stabilised by the catalytic hydroxide, its positive charge enhances the polarisation of the scissile peptide bond, making it more attractive for nucleophilic attack without the need for a strong oxyanion hole.

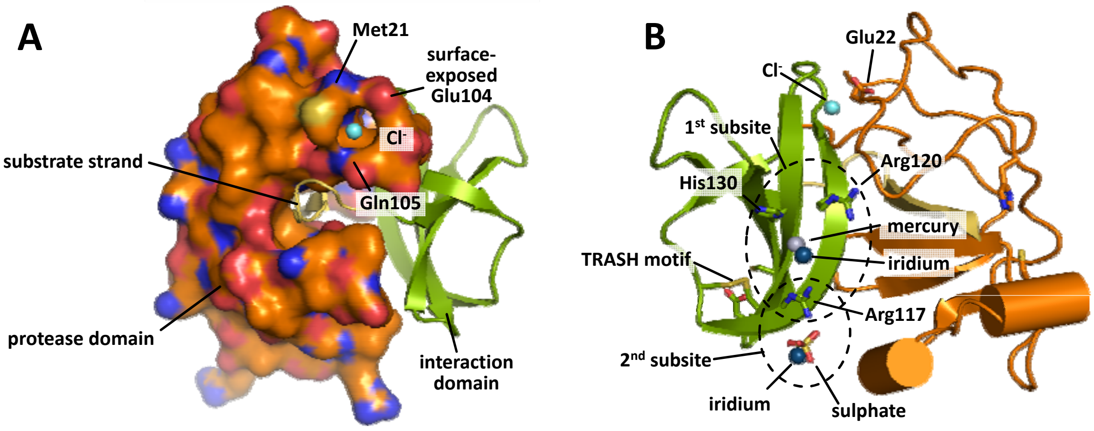

Figure S2: Specific ion binding in Npro identifies potential protein interaction sites, related to Figure 7.

(**A**) Identification of an electrostatic interaction site within the protease domain (orange) close to the N-terminus by chloride binding present in both Δ(2-21)Npro and Δ(2-21)Npro $S169P_{6His}$.

The pocket is located between the backbone amide of Glu22 and the side chains of Glu104, Gln105 and Tyr98. Three water molecules coordinated the Cl^- sitting in a solvent-accessible channel. By soaking crystals in NaCl-free buffers, we observed that the surface-exposed, negatively charged Glu104 side chain replaced the Cl^- ion and thus markedly changed the local surface and its charge distribution. These changes propagated in a 90° side chain flip of Asn105 and influenced stability and conformations of surrounding residues such as Ser124, Asn157 and the surface-exposed Val158 as seen in reduced electron density for the side chains of these residues. This may be an indication that the pocket serves as a protein-protein interaction site attracting negatively charged residues.

(**B**) Mercury (dark grey), iridium (dark blue) and ammonium sulphate (yellow sticks) bound to Npro reveal two electrostatic interaction clusters anchored at the Arg117 side chain.

Soaking experiments with ammonium sulphate, iridium hexachloride and mercury dichloride revealed an electrostatic interaction cluster within the I-domain composed of two neighbouring subsites. The first subsite was located in between residues Arg117, Arg120 and His130 forming a polar patch that was occupied by hydrated mercury chloride or iridium chloride complexes ($HgCl_2$ / K_2IrCl_6). The second subsite was located within 5 Å distance to the first cation binding site and was bound by hydrated iridium chloride complexes or sulphate. This site is formed by the side chains of Arg117 and Lys116 together with the backbone amide Arg117 and the adjacent His40 and Asn127 on the symmetry-equivalent molecule in the crystal. Both ion binding sites share Arg117 as a central ligand and thus underline its significance for protein interaction at the I-domain.
